# Complementary and alternative medicine utilization by a sample of infertile couples in Jordan for infertility treatment: clinics-based survey

**DOI:** 10.1186/1472-6882-13-35

**Published:** 2013-02-16

**Authors:** Sanaa K Bardaweel, Mayadah Shehadeh, Ghadeer ARY Suaifan, Maria-Vanessa Z Kilani

**Affiliations:** 1 Department of Pharmaceutical Sciences, Faculty of Pharmacy, The University of Jordan, Queen Rania Street, 11942, Amman, Jordan

**Keywords:** Complementary and alternative medicine, Infertility treatment

## Abstract

**Background:**

Although there is little information available to quantify the use of complementary and alternative medicine (CAM), growing evidence suggests that CAM prevalence among patients seeking infertility treatment is increasing worldwide. There are many products available on the market and many infertile patients demand information about CAM from their health care providers. This paper investigates the prevalence of CAM use among infertile couples in Jordan. Additionally, trends and factors contributing to CAM use for infertility treatment among these couples have been evaluated.

**Methods:**

A face-to-face questionnaire inquiring demographic information, use of CAM for medical conditions, in general, and types of CAM used for infertility treatment, in specific, was completed by one thousand twenty one infertile patients attending at two types of facilities; *in vitro* Fertilization (IVF) centers at both public and private hospitals and infertility private clinics. Both types of facilities were distributed in different areas of Amman, the capital city of Jordan. The study was conducted between May and August 2012.

**Results:**

Our results show that CAM therapies for infertility treatment were encountered in 44.7% of the study sample. The vast majority of CAM users were females. The most commonly used CAM therapies were herbs and spiritual healing. A clear correlation between the use of CAM for infertility versus the use of CAM for other chronic medical conditions has been found.

**Conclusions:**

The prevalence of CAM use for infertility treatment in Jordan is relatively high, particularly among young females, well educated and with a low income, in consistence with the studies reported elsewhere. Herbs and spiritual healing are widely used among patients in adjunct to conventional medical interventions. As CAM use is prevalent among patients, there is a clear need for health providers to become more aware of this phenomenon and for further research in this field.

## Background

Complementary and alternative medicine (CAM) was defined by the Cochrane Collaboration as “a broad domain of healing resources that encompasses all health systems, modalities, and practices and their accompanying theories and beliefs other than those intrinsic to the politically dominant health systems of a particular society or culture in a given historical period”. Recently, there has been a global interest in the use of CAM as a health care option. World Health Organization (WHO) and other studies have reported that more than three-quarters of the world’s population rely upon complementary and alternative medicine (CAM) for health care [1-4]. In 2007, adults in the United States spent $33.9 on CAM therapies and made 354 million visits to CAM practitioners [5]. The prevalence of CAM in developing countries is often connected to cultural beliefs and practices that lead to self-care, home remedies or consultation with traditional and religious healers [6-8]. Additionally, dissatisfaction with outcomes associated with conventional medicine [9,10]; and the apparent acceptance of naturalness and harmlessness of CAM [11,12] largely contribute to the wide prevalence of CAM in developing countries.

Existing definitions of infertility clearly show heterogeneity of criteria used to define infertility, rendering comparisons in prevalence of infertility among populations or over time extremely challenging [13]. In addition, the absence of a clear definition compromises clinical management and undermines the impact of research findings. For example, demographers tend to define infertility as childlessness whereas, the epidemiologists' definition is based on ‘trying for’ or ‘time to’ a pregnancy, generally in a population of women exposed to the risk of conception [13].

Infertility is estimated to affect 10–15% of couples in industrialized countries [14], however, the reported wide-ranging estimates of infertility prevalence can be explained by the versatility in how infertility is defined and measured. While the types of complementary therapies used during pregnancy have been reported [15,16], there has been little information about CAM used for infertility treatments worldwide [15,17].

Over the past 10 years, there has been a significant increase in the use of assisted reproductive technologies in Jordan. Nevertheless, little is known about the overall prevalence of infertility among Jordan’s population. To the best of our knowledge, literature search did not reveal any study that explored the use of CAM for infertility treatment in Jordan. Interestingly, various non medical therapies for infertility treatment, including spiritual healing, are commonly practiced among the public in Jordan.

The present study was planned to gain insights into the prevalence and factors leading to the use of CAM among infertile couples in Jordan. Additionally, the study was designed to bring to light CAM therapies which are most commonly used by infertile patients in Jordan. The study, also, aimed to investigate possible efficacy of CAM reported by infertile patients during their treatment. Moreover, the study was purposely designed to identify the main sources of information recommending the use of CAM for infertility treatment.

## Methods

Data was gathered from patients seeking infertility treatment in two types of facilities; i*n vitro* Fertilization (IVF) centers at both public and private hospitals and infertility private clinics. In the private clinics, patients were seen for routine investigation and treatment of infertility without any conception assistance being provided. Patients at IVF centers were provided with conception assistance. Both types of facilities were distributed in different areas of Amman, the capital city of Jordan.

Interviews were conducted during week days at different working times and included both genders of different age groups to assure sample homogeneity. The study was based on a structured questionnaire designed to fulfill the study aims. The interviews were conducted by fifth year pharmacy students who were trained with questionnaire administration and interviewing skills at The University of Jordan. The study took place between May 2012 and August 2012. Only patients (irrespective of their nationality) complaining of failure to conceive and attending any of the study centers were invited to complete the questionnaire. Verbal informed consent to participate in the study was obtained based on a standard written statement. Ethical approval for conducting the study was obtained from the Institutional Review Board (IRB) at the Jordan University Hospital (JUH) and the Scientific Committee at the Deanship of Scientific Research at The University of Jordan.

### Study population and study tool questionnaire

A face-to-face anonymous structured questionnaire was undertaken with one thousand twenty one infertile patients visiting any of the study centers (see Additional file 1). The target study sample was then divided into two groups, Group A and Group B. Group A included patients reported the use of CAM as infertility treatment aid and Group B included patients who did not use CAM during their treatment course. Subjects were recruited into the two groups based on their responses to the following question: “Have you ever used CAM for your infertility condition”. Additionally, a list of CAM therapies was provided in the questionnaire and the subjects were asked if they have ever tried any of the listed options for their infertility condition. Only those who responded with YES and selected at least one CAM option from the list were recruited into Group A. The questionnaire was structured by the study team based on preliminary discussions with patients and health professionals. Furthermore, the questionnaire was approved by a committee whose members were health professionals, consisting of two fertility specialists, a pharmacist and a nurse. The questionnaire was written in English and then translated into Arabic and back to English. Back translation of the questionnaire was undertaken to ensure its relevance and clarity, as well as to provide correlated versions of the questionnaire ready to be handled to the study population. Both versions of the questionnaire were checked by three members of the public with no medical background.

The questionnaire comprised of a total 18 questions and was divided into 3 sections. The first section investigated patients' demographical information that was expected to influence their health-related attitudes. In this part, patients were asked about age, gender, education, and income level. In the second section, patients were asked about their medical and infertility history. The third section involved questions inquiring about patients' knowledge, attitude and belief about CAM usage in disease conditions, in general, and in infertility treatment in particular. Additionally, patients were asked to specify which CAM therapies were used as fertility aid and, if applicable, their source of information for such therapies.

### Data analysis

Data was coded, entered and analyzed using Statistical Package for Social Sciences program (SPSS) database for Windows, version 17. The analysis of answers involved descriptive quantitative statistics e.g. frequency and percentage. Chi-square and Fisher exact tests were used to test for significant association between groups (p < 0.05).

## Results and discussion

The current study is a timely investigation of the Jordanian national profile use of CAM for infertility treatment. Jordan is a well known country for its population heterogeneity. Different nationalities, such as, Palestinian, Iraqi, Syrian, Lebanese, Egyptian, Libyan and Circassian are well mixed in the Jordanian community. This indeed ensures the versatility of the study place. A total of 1021 infertile patients (only infertile individuals were allowed to complete the survey) were interviewed after obtaining verbal informed consent to participate in the study. Our sample consisted of 74.7% females and 24.9% males. Unfortunately, 5 participants were unclear in identifying their gender and therefore; were excluded from our analysis. Table 1 summarizes the demographic characteristics that were anticipated to affect the use of CAM by infertile patients in Jordan.

**Table 1:** Characteristics of infertile patients participating in the study, Amman, Jordan Summer 2012 ( N=1021)

**Characteristic**	**Number**	**Percentage**
**Age (N= 1019)**^**a**^		
18–25	166	16.3
26–30	266	26.1
31–35	237	23.2
36–40	195	19.1
41–45	103	10.1
over 46	54	5.1
**Gender (N= 1017)**^**a**^		
Male	254	24.9
Female	763	74.7
**Education (N= 1012)**^**a**^		
Primary school	26	2.5
High school	222	21.7
Community college	179	17.5
Bachelor Degree	494	48.4
Postgraduate Degree	91	8.9
**Income per month JD**^**b **^**(N= 999)**^**a**^		
Less than 500 JD	261	25.6
500–1000 JD	414	40.5
1000–1500 JD	176	17.2
1500–2000 JD	74	7.2
More than 2000 JD	74	7.2
**Marriage period (N= 1004)**^**a**^		
Up to one year	50	4.9
1–2 years	162	15.9
2–4 years	225	22.0
More than 4 years	567	55.5
**Period trying to conceive (N= 917)**^**a**^		
Less than 6 months	154	15.1
6 months-1 year	171	16.7
1–2 years	201	19.7
2–4 years	181	17.7
More than 4 years	210	20.6

^a^Total number <1021 due to unanswered questions/missing data.

^b^Jordanian Dinar (JD 0.71 =USD 1.0).

CAM utilization for infertility treatment was encountered in 44.7% of patients. Compared to the prevalence of CAM among infertile couples reported in different parts of the world, a lower percentage (29%) was reported in a sample of 428 infertile couples in Northern California [18]. On the contrary, higher utilization percentages, 66% and 82% were reported by 100 infertile couples in South Australia [19] and 100 Turkish women in Turkey [20], respectively. Apparently, the variation observed may be attributed to cultural and socioeconomic differences between countries. Furthermore, expanded recognition and availability of CAM in developing countries, such as Jordan, might contribute to the relatively high prevalence of CAM for infertility treatment.

**Figure 1:**
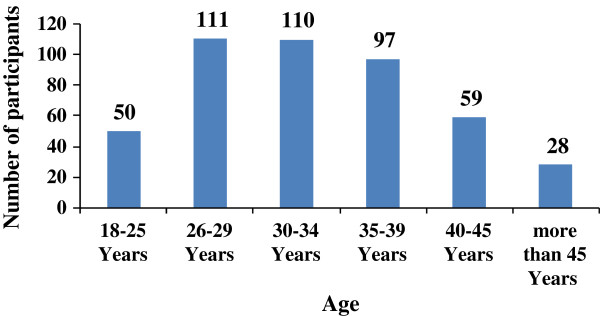
Prevalence of current CAM use for infertility treatment among participants by age group.

By and large, our study participants were relatively well educated; Bachelor degree holders (48%), and of low to intermediate economical level (500–1000 JD), as shown in Table 1. Notably, younger generations (range 18–34 years) formed 44.5% of the participants who used CAM as infertility treatment aid; Group A (Figure 1). Additionally, our results demonstrate that almost seventy percent (68.9%, CI 308/447) of Group A participants were of low to intermediate (up to 1000 JD) monthly income. These results are highly consistent with relevant studies that revealed similar profiles of CAM utilization for infertility treatment in terms of age and income level. According to a Turkish study, the mean age of CAM users, who utilized CAM for infertility treatment, was 26.7 years [20], however, this is different from the United Kingdom survey results where the median age was 35 years [21]. Nevertheless, our results are congruent with the Nachtigall’s report which indicates that low-income infertile Latino couples frequently utilize traditional medical remedies (such as teas and massage) for their infertility condition [22]. This suggests that infertile patients tend to utilize CAM as a lower cost treatment alternative [23]. Taken together, one may presume that older patients (> 40 years old), with correspondingly higher incomes (> 2000 JD), are more likely to afford the cost of conventional medical interventions for their infertility treatment, hence their poor interest (40.0%, 8/20) in CAM as an option for infertility treatment.

**Figure 2:**
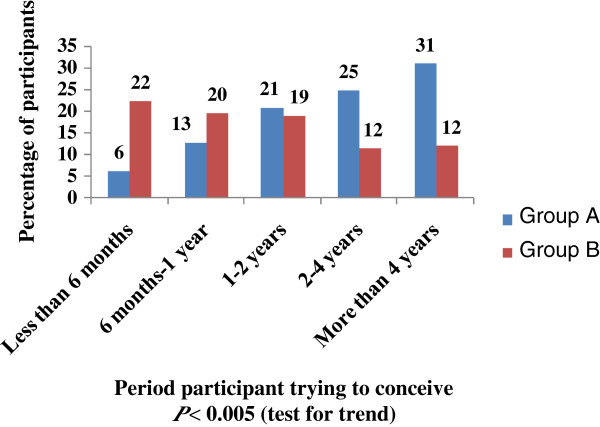
Participants trying to conceive by period of time, P < 0.005 (test for trend).

In our study sample, the tendency to use CAM for infertility treatment significantly (p < 0.005) increased as the years of marriage increased, as 62.4% of Group A participants were married for more than 4 years. Interestingly, during marriage period, contradictory concern between Group A and Group B towards conceiving was observed. Group A willing to conceive appears to decrease gradually during marriage (as clearly shown in the last 6 month period of their marriage time), whereas, group B curiousness increases. This observation is illustrated in Figure 2. To date, studies on CAM as a treatment option for infertility have demonstrated benefit [24] , ineffectiveness [25] and harm [26]. Although there is little evidence supporting their effectiveness to treat infertility, the demonstrated role of CAM in infertility treatment could be attributed to its psychological effect. Based on our results, we found that women who used CAM were more distressed and so emotionally affected by their fertility problems than non-users, this indeed might result in lowering their interest to conceive. However, from our design, we cannot ascertain the exact clue for the deduction observed in Group A willing to conceive. Therefore, further studies on possible physiological and/or psychological effects of different CAM interventions, used for infertility treatment, should be evaluated in our future work research.

**Figure 3:**
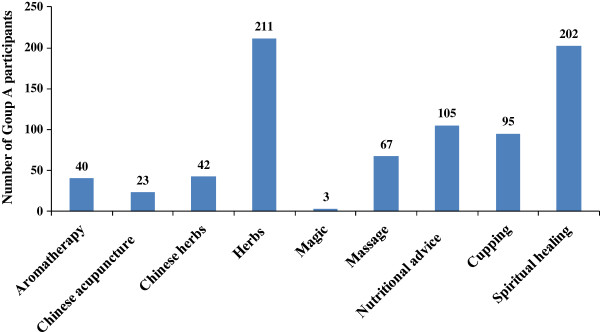
Common CAM therapies used by infertile patients in Jordan.

According to our study, religious healing, which was encountered in 43.8% (CI 34.5% to 53.0%) of Group A, along with herbs, used by 61.6% (CI 56.3% to 66.9%) of Group A, appear to be the most widely used regimen for infertility treatment (Figure 3) in Jordan. These findings were in good agreement with the Turkish [20] and the United States studies [18], which have reported that religious healing and herbs were the most commonly utilized modalities for infertility treatment. In Jordan, likewise in other Middle East countries, religion has strong influence on peoples' beliefs about illness and treatments resulting in acceptance of methods recommended by religious healers without rational criticism.

Almost one of every five participants of Group A (16.4%, CI 6.1% to 26.8%) was diagnosed with other chronic medical conditions. An evident correlation between the use of CAM for infertility and the use of CAM for other chronic medical conditions was found. This is supported by the observation that 82.7% (CI 77.5% to 87.9%) of those who used CAM for infertility treatment also used CAM for the treatment of their other medical conditions.

Generally, the common belief in herbs safety is largely attributed to an obvious misconception; based on the fact that herbs and herbal products come from ‘nature’ and are therefore ‘naturally safe’ [12,27] or ‘intrinsically harmless’ [27]. Scientific literature is rich in studies indicating potential sensitizing capacity, organ toxicity and carcinogenic properties of many herbal remedies. In addition, mechanical injuries and infectious complications that result from acupuncture were also reported in literature [28,29]. Our results indicate that Group A participants fairly trusted CAM therapies, generally, and herbal products, precisely, to be safe. Almost two thirds of Group A participants (63.8%, CI 59.6 to 68.0%) did believe in CAM safety for the treatment of their chronic medical conditions as well as for their infertility. As apparent, only 46.5% (CI 41.6% to 51.5%) of Group A participants were aware of potential CAM side effects. Nevertheless, higher awareness of possible side effects was characterized in Group B participants, 67.7% (CI 64.7% to 70.7%).

**Figure 4:**
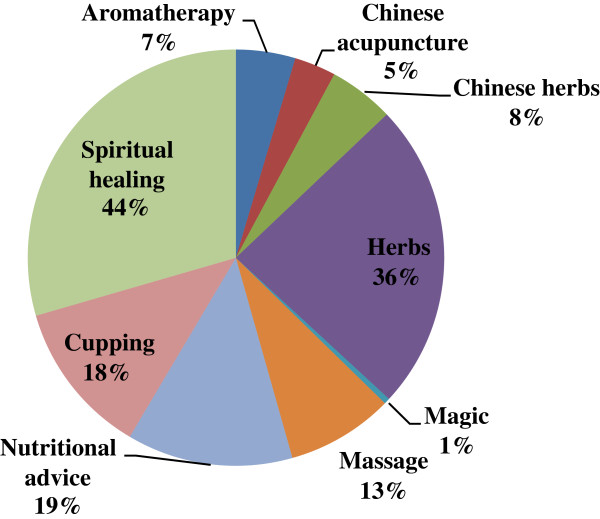
Usage patterns of CAM therapies among Group A; respondents supporting the helpful effect of CAM in their infertility treatment.

As indicated in this study, herbal medicine is the most widely trusted and employed form of CAM therapies being used by 61.6% (CI 56.3% to 66.9%) of Group A participants. This trend is supported by the patients' belief that herbs are safer than conventional medications and may also reflect greater access to herbs, which have relatively high availability in Jordan. Furthermore, more than one third of Group A participants (36.1%, CI 25.2% to 47.0%) considered herbs to be effective in their infertility treatment (Figure 4).

Nutritional advice and cupping therapy are commonly used in traditional eastern medicine. Many of these non medical interventions have existed for generations, and still in use for infertility treatment as well as for other chronic medical conditions. In our study, 23% and 21% of Group A participants reported the use of nutritional advice and cupping therapy for their infertility treatment, respectively. Interestingly, of patients who had used nutritional advice or cupping therapy, approximately 19% thought it had been helpful for their infertility treatment. Given that such non medical therapies, identified by this study for infertility treatment, are still in use, usefulness or efficacy of these interventions may indeed exist.

**Figure 5:**
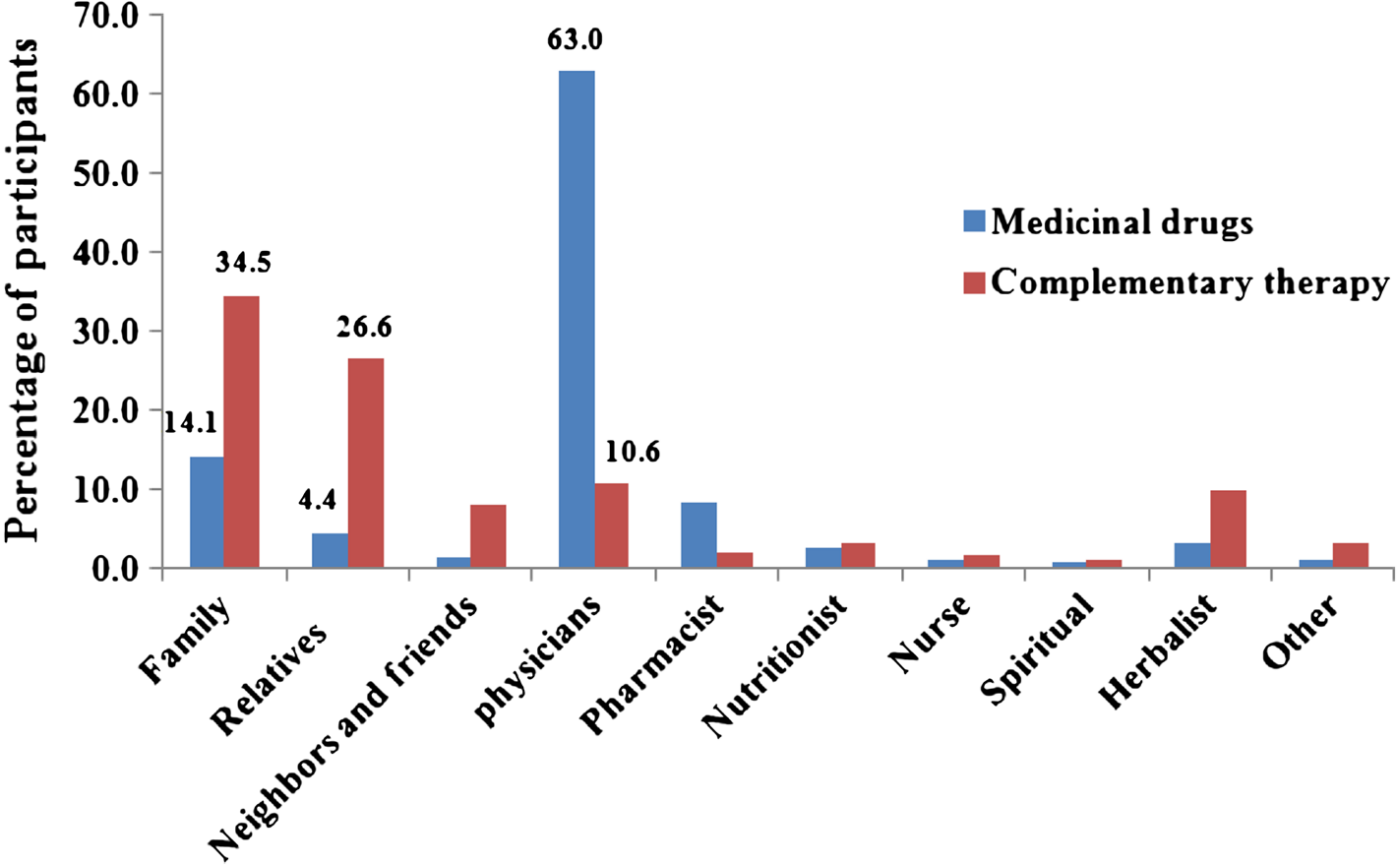
Information and recommendations sources of CAM for infertile patients in Jordan.

According to our results, the main sources for CAM recommendations were the family (husband, wife, sons, daughters, sisters and brothers), relatives (grandfather, grandmother, uncle, aunt, nephew, niece, etc.), followed by herbalists, while very few recommendations were made by the physicians and pharmacists, as shown in Figure 5. On the contrary, physicians were the main source for medications used for infertility treatment. In accordance with the results of other studies [12,30-32], although several CAM options and approaches are frequently used in conjunction with conventional medical treatments, information about CAM use is generally not inquired by physicians nor provided by them to their patients. In addition, it is worth mentioning that only one in every three participants (32.7%, CI 13.6% to 40.2%) who were advised to use CAM for infertility treatment by their physicians, did receive alert about CAM possible side-effects or risks from the physicians.

Previous studies based on CISCOM Complementary Medicine database regarding the use of CAM for infertility and miscarriage were generally poor, not well structured, and with no study provided prospective randomized controlled evidence of clinical efficacy for any form of CAM to improve the prognosis for infertile patients [21]. To our knowledge this is the first reported study regarding CAM use among infertile patients in Jordan. Further studies on minority groups and from other geographic regions will further illuminate factors associated with patients' interest in CAM therapies for infertility treatments. Additional well-designed and controlled studies will be required to demonstrate whether CAM treatments are likely to be of benefit or determinant to individuals seeking treatment for infertility.

## Conclusion

This study provides results of a survey of infertile couples in Jordan (n = 1021), seeking medical assistance for their infertility condition. Our data suggests a high use of CAM, particularly, among young females, well educated and with a low income. By and large, religious healing and herbal therapies are the most widely CAM therapies used among infertile patients in Jordan. The growing prevalence of CAM use among infertile patients in Jordan necessitates further research of this phenomenon.

## Competing interests

The authors declare that they have no competing interests.

## Authors’ contributions

SB participated in the design of the study, acquisition and interpretation of data, helped to draft the questionnaire, participated in writing the manuscript and revised it. MS participated in the design of the study, helped to draft the questionnaire and participated in writing the manuscript and revised it. GS discussed and evaluated the results, performed the statistical analysis and participated in writing the manuscript and revised it. MK participated in the design of the study and helped to draft the questionnaire. All authors read and approved the final manuscript.

## Pre-publication history

The pre-publication history for this paper can be accessed here:

http://www.biomedcentral.com/1472-6882/13/35/prepub

## Supplementary Material

Additional file 1 Questionnaire on Complementary and Alternative Medicine Utilization for Infertility Treatment. Click here for file
