# Supplementary material for: Complementary and alternative medicine utilization by a sample of infertile couples in Jordan for infertility treatment: clinics-based survey
Source: BMC Complement Altern Med. 2013 Feb 16;13:35. doi: 10.1186/1472-6882-13-35 (PMC3599001; doi:10.1186/1472-6882-13-35)
Supplement: Additional file 1 — Questionnaire on Complementary and Alternative Medicine Utilization for Infertility Treatment. [file 1472-6882-13-35-S1.pdf]

Form Number:

Complementary and Alternative Medicine Utilization by a Sample of Infertile Couples in Jordan for Infertility Treatment: Clinics-Based Survey

May 2012-August 2012

Any information collected through surveys will be used in reports only on an aggregate basis. That is, it will not be possible for anyone to identify a particular individual with any set of responses. We strive to ensure that data is kept secure, and that we collect only as much personal data as is required for the survey. We assure you that your responses will be kept confidential.

Please ticks (X) in the appropriate answer box □or fill in the provided space for the following questions:

1. **Gender** □ Male □ Female
2. **Age** □ 18-25 □ 26-30 □ 31-35 □ 36-40 □41-45 □over 46
3. **Education level** □ Primary school □High school □Community college □Undergraduate studies □Postgraduate studies
4. **Monthly income of the family** □Less than 500 JD □500-1000 JD □1000-1500 JD □1500-2000 JD □ More than 2000 JD
5. **How long have you been married?**
   □Up to 1 year □1-2 years □ 2-4 years □More than 4 years
6. **How long have you been trying to conceive?** □Less than 6 months
   □6 months-1year □1-2 years □2-4 years □More than 4 years
7. **Have you ever been diagnosed with any chronic medical condition?** □Yes □No
   If your answer to Q7 was Yes, please answer Q8; if NO, please move to Q9
8. **Have you ever used complementary therapies for the treatment of your chronic medical conditions?** □Yes □No
9. **Have you ever used complementary therapies for the treatment of your infertility condition?**
   □Yes □No
10. **If you used complementary therapies for infertility treatment, then where did you get the recommendations for the utilization of such therapies?**
    □Family □ Relatives □Neighbors and friends
    □General physician □Pharmacist □Nutritionist □Nurse □Spiritual
    □Herbalist □Other (mention………….)

11. If you used complementary therapies for infertility treatment, then which of the following regimens did you use?

□Aromatherapy □Chinese acupuncture □Chinese herbs
□Herbs from the herbalist □Magic □Massage □Nutritional advice
□Cupping □Spiritual healing □Other (specify……..)

12. Was the complementary therapy used for your infertility condition helpful?

□Yes □No

13. Do you believe that complementary therapies, in general, are safe to be used for infertility treatment and for other chronic medical conditions? □Yes □No

14. Do you believe that herbs, in particular, are safe to be used for infertility treatment and for other chronic medical conditions? □Yes □No

15. Were you concerned or notified about any possible side-effects attached to the complementary therapies used for your infertility condition treatment?

□Yes □No

16. If you have been recommended to use complementary therapies for infertility treatment by your physician, have you been alerted about possible side-effects?

□Yes □No

17. Have you ever used medicinal therapies for the treatment of your infertility condition?

□Yes □No

18. If you use medicinal therapy for infertility treatment, then who do you usually consult?

□Family □Relatives □Neighbors □Physician □Pharmacist □Nutritionist
□Nurse □Spiritual □Herbalist □Other (mention………………..)
